# What You May Not See Might Slow You Down Anyway: Masked Images and Driving

**DOI:** 10.1371/journal.pone.0029857

**Published:** 2012-01-18

**Authors:** Ben Lewis-Evans, Dick de Waard, Jacob Jolij, Karel A. Brookhuis

**Affiliations:** 1 Traffic and Environmental Psychology Group, Neuropsychology, University of Groningen, Groningen, The Netherlands; 2 Vision and Cognition Group, Experimental Psychology, University of Groningen, Groningen, The Netherlands; 3 Technology, Policy and Management, Delft University of Technology, Delft, The Netherlands; The University of South Wales, Australia

## Abstract

Many theories of driver behaviour suggest that unconscious or implicit emotions play a functional role in the shaping and control of behaviour. This has not been experimentally tested however. Therefore, in this study the effects of emotive masked images on driver behaviour were examined. While driving a simulator, participants were repeatedly exposed to negative or neutral emotionally laden target images that were sandwich masked by emotionally neutral images. These images were encountered across two different trials each of which consisted of 3–4 minutes of driving on a rural road. The results indicate an effect of the negative target images primarily in reducing the extent of familiarisation occurring between the first and second experimental drives. This is evident in a reduced decrease in heart rate and a reduced increase in high band heart rate variability and actual travelling speed from the first to second drives if the negative target image was presented in the second drive. In addition to these findings there was no clear effect of the target image on subjective ratings of effort or feelings of risk. There was however an effect of gender, with the majority of the effects found in the study being limited to the larger female dataset. These findings suggest that unconscious or implicit emotional stimuli may well influence driver behaviour without explicit awareness.

## Introduction

Many recent models that seek to explain driver behaviour have come to incorporate functional views on the role of emotions in driving. These theories include Task Difficulty Homeostasis theory (TDH) [1], [2], Risk Allostasis Theory (RAT) [3], the Multiple Comfort Zone Model [4], the Risk Monitor Model (RMM) [5]–[7], feeling of risk homeostasis [8], and the situational control framework [9]. The way that emotions are said to effect driving differs somewhat between the models, for example in the Multiple Comfort Zone Model [4], [10] they operate in a threshold manner with emotions being produced by breaches in safety margins. This is in contrast to the account given by RAT [3] or RMM [5]–[7] where levels of preferred emotions or feelings are set and constantly monitored. However, as mentioned above, all the models view emotions in a functional fashion, suggesting that they play an important role in biasing or influencing driver decision making, sometimes even without entering into explicit conscious awareness. So for example uncomfortable emotions, such as those related to the risk arising from a narrow road, would signal to the driver to be cautious and to reduce their speed. RMM in particular takes a strong functionalist stance suggesting that risk, and the detection of risk via emotions and feelings, is a vital evolutionary adaptation for survival [5]–[7].

Most of the above models approach the issue of emotion via reference to the Somatic Marker Hypothesis [11], [12]. The Somatic Marker Hypothesis states that emotions, defined as unconscious physiological states, and feelings, which represent the later conscious awareness of these emotions, have a significant impact on human decision making. In particular unconscious emotional physiological states are presumed to arise in reaction to certain learnt or innate stimuli, and ‘mark’ such stimuli or situations in ways that bias decision making towards or away from them. The presence of these emotional physiological states can typically be detected via psychophysiological measurements.

The main experimental evidence for the Somatic Marker Hypothesis comes from a study carried out using the Iowa Gambling Task that compared neurologically ‘normal’ people with patients who had impaired emotional systems due to ventromedial prefrontal damage [13]. The Iowa Gambling Task involved participants losing or gaining fake US currency by drawing from four decks of cards. Two of the decks had high gains but also high losses, making them poor choices in the long run, and the other two had smaller pay outs but also smaller losses, making them a better long term choice. What was found is that even before the ‘normal’ participants could report that they consciously knew which decks were the best they were producing detectable changes in skin conductance when choosing a card from a poor choice deck. This was taken as a sign of somatic, or body, markers being formed, which predated conscious awareness and eventually helped the participants to decide which deck was the best decision. The skin conductance response was missing in the impaired individuals and they continued to select cards from the poor decks. This particular interpretation of the Iowa Gambling task has been challenged however, with some researchers suggesting the results obtained, at least in terms of the deck choice performance, could better be explained via problems with working memory, attention, or rehearsal learning in the impaired individuals [14], [15].

Outside of the Somatic Marker Hypothesis, the majority of the above driver behaviour models also owe some of their structure in terms of how they refer to emotions to the work of Taylor [16]. Taylor claimed to show that skin conductance reacted during a drive at areas of high accident occurrence. Furthermore Taylor claimed that over time skin conductance levels were kept relatively stable, and suggested that this meant that drivers were targeting or trying to maintain a set level of anxiety or risk while driving. This same targeting view is taken by TDH [1], [2], RAT [3] and RMM [5]–[7] who all reference Taylor's findings in terms of the consistency of skin conductance responses. This view was also influential on earlier models of driver behaviour such as Risk Homeostasis Theory [17], [18]. The idea of skin conductance response consistency is not universally accepted however. In particular, Taylor's findings have been challenged on the grounds that skin conductance is a quite reactive and relatively non-specific measure. Meaning that skin conductance responds to many other factors in addition to, or instead of, emotional changes [1], [10], [19], [20]. For instance, it is possible that Taylor's findings in terms of skin conductance could be explained as simply arising through the motor control of the vehicle required for driving [10] rather than reacting to any changes in, or reflecting the maintenance of, emotional or risky elements. The Zero Risk theory of driver behaviour [21], [22], and the later Multiple Comfort Zone Model [4], [10], have also challenged the idea of maintaining and targeting a constant level of risk or anxiety. These two models instead argue that most of the time drivers experience, both consciously and unconsciously, no risk or anxiety when driving, and that when it is experienced it acts as a warning to change behaviour, unless drivers are otherwise motivated to accept the experienced risk.

Putting aside the differences between the particular theories, there is no doubt that driver behaviour models are trending towards a functional role of emotion and feelings in the control of driving, particularly when it comes to risk judgment. This trend in traffic psychology is a reflection of a wider trend in psychology where the functional importance of emotions and feelings in decision making is being stressed [1], [3], [7], [23]. For example in Slovic et al. 's [24] view of risk assessment there are ‘affect heuristics’ which are fast and automatic emotive reactions to risky situations that can be used in guiding decision making. This affect based system is contrasted to the more traditional, analytic, and subjective utility maximization view of risk assessment, which can still operate in certain situations. However, the ‘affect heuristic’ is hypothesized to be commonly used in day-to-day decision making.

While the idea of implicit or unconscious emotional effects on decision making has become popular, it presents an interesting challenge to experimentally test in a complex task like driving. Exactly how can an emotion be generated in a participant without it entering into awareness and becoming a feeling? And then, how can its impact be tested without again explicitly alerting the participant?

One possible solution to this challenge is the use of masked images. This is where emotionally charged images are very briefly shown to participants with other, longer lasting images shown before and after the emotionally charged image in order to ‘mask’ it from the participant's conscious perception. The idea is that the image can still generate an emotive response, but it does so without entering into the conscious awareness of the participant, or at least without the participant becoming explicitly aware of it. This process is also referred to as priming, in that the target images used are associated with situations that prime or trigger certain emotions, feelings, and cognitions in individuals via preconscious processing. In the case of this study for instance it was hoped that the negative images used would prime reactions in the physiology of participants and lead to slower speeds and faster reaction times to road safety relevant stimuli, such as a stop sign. The idea that emotionally negative images can be processed preconsciously is well in line with functional thinking in terms of emotions and feelings, in that it would be potentially evolutionary advantageous to react to threatening or emotionally negative situations as fast as possible. However, it should also be noted that whether masked images are truly unconscious, or subliminal, is a matter of great debate [25]–[27]. As such, they will be referred to as simply masked, rather than subliminal in this paper.

Using masked images has certain merits. Past studies have shown that emotionally negative masked images produce skin conductance responses [28]–[30] and activate areas of the amygdala that are in accordance with fear or threat detection [31]–[34] without being able to be reported as perceived by participants. Further evidence for the emotional influence of unconscious images comes from research on patients with blindsight. Blindsight is a condition where due to damage to the visual cortex individuals are unaware of visual stimuli but still retain a limited ability to make judgements about visual aspects of the world around them [35]. Research with blindsight patients, or with individuals in which blindsight has been induced [36], has shown that they still have some ability to detect visual emotional stimuli, and that these stimuli activate relevant fear or threat detection areas of the amygdala despite not entering into explicit conscious awareness [36]–[38].

Masked images have also been shown to impact on attitudes and judgements about others [39], as well as the level of hostile behaviour performed towards them [40]. Additionally, masked images have been found to affect risky decision-making based on masked monetary rewards in simple gambling like experimental tasks [41], [42]. There is also some evidence that masked images can affect attention and mental workload. For example Carlson, Fee, and Reinke [43] found that participants would react faster on a dot-probe task when the dot occurred on the same side as a threatening masked image. In other research [44] it has been found that presenting masked images showing death and mutilation or physical threats (such as a growling dog or striking snake) increased gaze duration towards the images that showed the physical threat, and decreased gaze duration towards the images that simply contained physical injury or death.

Ultimately the above studies suggest that masked images can have some influence on an individual's decision making or attention. However, these studies have been carried out with relatively easy tasks in relatively simple conditions, and we are unaware of any research examining the influence of masked images on a complex task such as driving.

Driving is generally viewed as a self-paced task [16], [21], [45], [46], in that drivers can to a large extent set their own pace of movement through the road system, and generally can alter and control the challenge of the driving task. This is mostly done through highly automated actions [10]. It is however a task that involves many individual drivers interacting within a large and varied road system, and with a wide range of regulative control. Navigating this complex system properly is important, because objectively speaking at most times drivers and other road users are only moments away from death or serious injury. A fact that is sadly well represented in road accidents being the leading cause of death for people aged 15–29 worldwide, and the 9^th^ leading cause of death overall across all age groups [47]. It is therefore important to develop a good understanding of what variables shape and affect driver behaviour.

The study described in this paper therefore sets out to experimentally test the influence of implicit or unconscious emotional signals on driver decision making, with a focus on driver speed choice. Speed is one of the most prominent road safety issues. Not only because of the influence of inappropriate speed choices on the chance of having an accident, but also because of the undeniable influence of velocity on physical trauma and property damage when an accident does occur [2], [48]. Speed is also one of the main ways in which drivers can regulate the task demands of driving, and therefore can ‘self-pace’ the driving task [16], [21], [45], [46].

Participants in the present study were asked to drive a simple rural road in a driving simulator while paying attention to a series of images presented just below the rear view mirror. The images were presented under the pretence of carrying out a memory task. Each participant drove the road twice, with one drive involving the presentation of emotionally negative target images and the other of emotionally neutral target images. These images were in both cases backwards and forwards masked by different emotionally neutral masking images. At some point these masking images would change to include a stop sign. When this occurred participants had to stop the car as quickly as possible. Information on speed and stopping time along with subjective impressions of effort and feeling of risk were collected alongside physiological measures of skin conductance, heart rate, and respiration.

In line with the functional account of emotion provided by the various models of driver behaviour discussed above [1]–[10] it was predicted that when participants were exposed to the negative target images they would drive at a slower speed than when exposed to the neutral target images. This was predicted to occur due to the production of uncomfortable emotions associated with the images, which signal to the drivers that something is amiss with their behaviour or the road environment and therefore leads them to take action to remove or reduce this emotion. Due to the use of masking these emotions should occur unconsciously or at least without explicit awareness, and therefore it is also hypothesised that any behaviour changes will occur without any meaningful change in risk or effort ratings between the two different target image conditions. In addition, in line with a physiological and functional account of emotion it was also predicted that the psychophysiological measures taken would show a significant response in line with a negative emotional reaction arising from the emotionally negative images. Finally, it was predicted that when participants were exposed to the negative target images that they would become more alert to potential road safety related stimuli and therefore respond faster to the stop sign image in bringing their vehicle to a stop.

## Methods

### 2.1 Participants and Ethics Statement

Ethics approval, including permission to deceive the participants, was gained from the University of Groningen Psychology Ethics Committee. Participants were informed that their information would be treated anonymously and that they could withdraw from the experiment at any time with no penalty. Participants were also debriefed at the end of the study, and the masking procedure was fully explained.

Participants were recruited through the English speaking University of Groningen participant pool and given course credit for participation. Participants were required to have held a valid car drivers licence for at least one year. This resulted in 74 females and 39 males being recruited for the study. However, one female reported feeling uncomfortable with the simulator during the practice drive and therefore did not progress in the study. In addition, two female, and six male participants reported being explicitly aware of the target images and were also removed from the sample. Furthermore, seven males and 12 females mentioned that they thought that perhaps there was an image being shown that they could not see even though they could not report what it was. In order to present results that are as conservative as possible in terms of the awareness of the target images these participants have also been excluded.

This results in a final sample size of 85 participants. The remaining 59 females were 21.09 years old on average (SD 2.07) and had held their licence for an average of 3.19 years (SD 1.97). The remaining 26 males were 21.62 years old on average (SD 1.83) and had held their licence for an average of 3.44 years (SD 1.41).

### 2.2 Materials

#### 2.2.1 Driving Simulator

The University of Groningen driving simulator was used in this study. It is a fixed base simulator running on STSoftware software©. The simulator consists of three high definition plasma screens, all set to a refresh rate of 60 Hz. The graphics engine of the simulator software itself runs at 60 frames per second, which was confirmed via the FRAPS© software package. In total the simulator provided participants with a 210-degree view of the road environment.

The road environment resembled a simple rural road with a consistent gentle s-curve in order to create some steering demand during the task. During all drives speed information was concealed through the use of a cardboard cut-out. This was to force participants to rely on their own perception of speed, rather than the speed provided by the speedometer. The simulated car was set to operate in automatic gear mode in order to minimise any movement related artefacts in the collection of the psychophysiological data.

#### 2.2.2 Images

The images used during the task were taken from the International Affective Picture Set (IAPS) [49]. The following images where used as negative target, neutral target, and masking images:

- Negative target images: IAPS numbers 3000, 3010, 3015, 3053, 3060, 3064, 3068, 3069, 3080, 3102, 3110, 3120, 3150, 3400, 9410, 9433, 9901, 9910, 9911, 9920
- Neutral target images: IAPS numbers 5130, 6150, 7000, 7009, 7010, 7020, 7030, 7037, 7040, 7050, 7060, 7080, 7090, 7190, 7217, 7234, 7235, 7500, 7700, 7705
- Masking images: IAPS numbers 7002, 7004, 7006, 7031, 7035, 7036, 7038, 7042, 7052, 7055, 7056, 7057, 7059, 7100, 7150, 7175, 7224, 7233, 7491, 7950

The negative target images mostly consisted of mutilated and deceased humans with some images of car accidents. According to the IAPS standardised scores, the negative images had valence ratings between 1.31 and 2.5 (mean 1.80), and arousal ratings between 5.70 and 7.26 (mean 6.59). The neutral target images and masking images consisted mostly of household items such as mugs, bowls, and forks, along with some pictures of buildings. The neutral target images had valence ratings between 4.23 and 5.55 (mean 4.86), and arousal ratings between 2.17 and 3.84 (mean 2.78).

**Figure 1:**
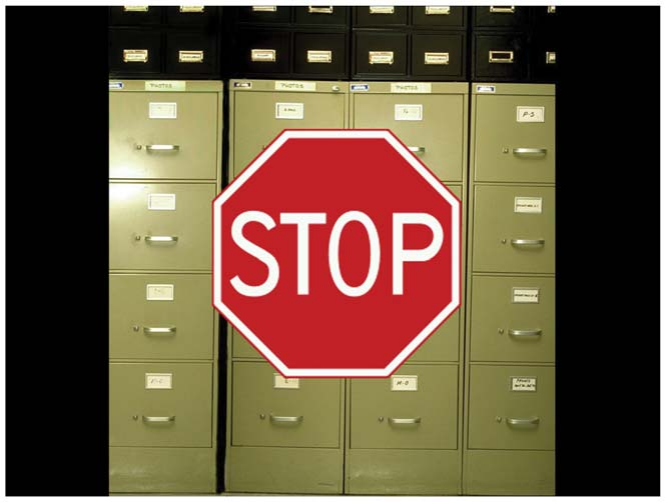
An example of a masking image with the stop sign added.

The images used for masking (the masking images) had valance ratings between 4.45 and 5.55 (mean 4.97), and arousal ratings between 1.72 and 4.02 (mean 2.76). It was these masking images that were used to forward and backwards mask the above neutral and negative target images during the trials. Variations of the masking images were also created for the reaction time task that had standard stop street signs placed in the centre of the images (see figure 1). All of the images were stored in JPG format, with a resolution of 256×192 pixels.

#### 2.2.3 Psychophysiological Measures

Participants were asked to wash and dry their hands and Tin (Sn) electrodes with some saline paste where taped to the distal phalanxes of the index and third finger of their left hand to measure skin conductance. This method of attachment is somewhat unusual, but has been used successfully in the past and allows for good control of steering without creating interference in the skin conductance measures [50]. Participants were also fitted with Polar© and Respitrace© belts in order to collect cardiovascular and respiration data. Profiles of skin conductance and respiration information were created using the Brain Vision Analyzer© software package, and mean skin conductance and respiration levels were calculated. Heart rate was processed via the CARSPAN software package, with each file also being visually inspected for artefacts and manually corrected if necessary [51]. Along with the collection of heart rate, spectral analysis was also run in CARSPAN to calculate heart rate variability in the high (0.15–0.40 Hz) and mid (0.07–0.14 Hz) frequency bands [52]. Finally Brain Vision Analyzer© was also used to calculate mean heart rate, and mean heart rate variability.

Due to the variation in the moment the stop sign was shown, the psychophysiological data was shortened to only account for the first 180 seconds of driving. Furthermore, there is an immediate effect of beginning to drive on all the physiological measures, so the first 30 seconds of data was also removed to eliminate any biasing effect that this may have caused. This left 150 seconds of data for use in later analysis. In addition four males and 16 females had distorted or missing psychophysiological data and had to be excluded from analyses, resulting in a sample size of 65 participants for the analysis of the psychophysiological data. The participants in this smaller sample did not significantly differ (p>0.05) in gender, age, licence status, speed of driving or any of the subjective measures from the larger 85 person sample, and therefore are assumed to have come from the same underlying population.

### 2.3 Procedure

**Figure 2:**
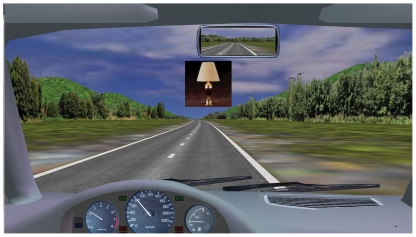
Screenshot of the simulator's centre screen. This shows the road environment and an example masking image in the position in which the images were presented during the trials.

Participants first filled out a demographic questionnaire to gather data on their age, gender, and driving experience. The experimental procedure was then described to them under the pretence of being a study about the effect of memory tasks on driving. Participants were told that they were to drive at whatever speed they found comfortable, but that while doing so they were to carry out a memory task. This memory task involved paying attention to constantly changing images that were presented in the upper centre of the screen (just under the rear view mirror as shown in figure 2).

The participants were told that they had to count the total number of times that the currently presented image was the same image that they had seen presented directly before. Participants were able to do this as there was a noticeable flash when each image was presented which signalled that a new image had appeared. So, if the participants saw a shoe, and then a shoe again, they would have to add one to the count of image couples that they had observed. However, if they saw a cup, then a chair, and then a cup they were to ignore that as none of the images were repeated directly after each other. They were told that they did not have to remember what the images were, only the total number of direct image repeats that they had seen during the drive and that they would have to write this down at the end of the trial. Participants were also told that at some point during the drive a stop sign would appear over top of the normal (masking) images and that when they saw this sign they were to stop counting repeats and bring the car to a full stop as quickly as possible.

The images that the participants were instructed to pay attention to were the masking images mentioned above, and were presented on the screen for 50 frames (800 ms). Unbeknownst to the participants in between each masking image a target image was presented for 2 frames (32 ms). With the timing of the image presentation confirmed through the use of a high speed digital camera over three four-minute periods during the initial setup of the experiment. The target images shown were either negative or neutral in emotive content depending on the trial. The presentation of the images began only once the participants had driven 100 metres, and continued until the participants brought the vehicle to a complete stop. After three minutes a random timer was started that triggered the presentation of the stop sign variant images to replace the masking images within 1–60 seconds.

The masking and target emotive images were selected randomly every time they were presented and programmed to usually avoid directly repeating themselves so that the same image would not often be shown twice in a row. However, this was allowed to randomly occur on occasion, creating the supposed ‘memory task’ for the participants when the masking images would repeat two times in a row.

Participants were asked to sit in the driving simulator and adjust the seat so that it was comfortable. They were then given a practice drive to get familiarised with the simulator and the memory task. During this practice task no data was recorded. The practice drive lasted 3–4 minutes, depending on the timing of the stop sign, and was identical to a neutral image experimental trial. Also at the start of the practice drive the experimenter verbally pointed out the first directly repeating image to the participant. This was done to make sure that the exact nature of the memory task was understood.

After the practice drive participants were asked if they felt comfortable with the simulator and the memory task. At this point all participants stated that they were comfortable and wished to continue. Then the participants were asked to hold the steering wheel, but to otherwise sit still and quietly while baseline physiological measures were taken. These baseline measures were then collected for 3 minutes.

After the baseline data collection period participants were asked to drive the road again twice, while carrying out the memory task. During these two drives data on travelling speed was collected at a rate of 10 Hz. Also the braking reaction time to the stop sign image was calculated as the time from when the first stop sign appeared until the participant had depressed the brake pedal by more than 5 percent. One trial for each participant involved the neutral target images and the other the negative target images. The order was counterbalanced across participants, however due to scheduling issues, and loss of participants due to the earlier mentioned incomplete data sets the end result was that 43 participants (32 female, 11 male) drove with the negative target image trial first and then the neutral, and 42 (27 female, 15 male) with the neutral target image trial first and then the negative.

After each trial participants were asked to fill in a questionnaire containing the following open ended questions:

1. How many times during the drive did you see paired presentations of images?
2. On average, what speed (in km/h) do you think you drove at during the last trial?
3. On average, what speed (in km/h) would you typically drive while following the route you just saw?

Then participants were also asked to provide a driving effort rating for the trial they had just completed on the rating scale for mental effort (RSME) [39]. The RSME is a unidimensional scale ranging from 0 to 150, with several unevenly placed anchor points along it going from ‘absolutely no effort’ at the bottom (a RSME score of 2) to ‘extreme effort’ near the top (a RSME score of 112). A modified version of the RSME was also used to assess feeling of risk, with the effort related anchors being replaced with the following risk related anchors; absolutely no risk, almost no risk, a little risk, some risk, rather much risk, considerable risk, great risk, very great risk, and extreme risk. After the second trial the participants were additionally asked the following two open ended questions which served as manipulation checks to make sure that the negative or neutral images had not been detected:

1. What was/were the difference(s), if any, that you noticed between the first and second roads you drove?
2. Did you notice any images during either drive that seemed out of place, unusual or particularly disturbing? If so, what were they?

Once participants had completed the practice drive and both experimental trials, the psychophysiological recording equipment was removed and they were fully debriefed about the use of the hidden, neutral and negative images in the experiment. This included an additional verbal check to see if they had detected the negative or neutral images, and an opportunity for participants to ask any questions that they may have had.

### 2.4 Analysis

Due to the variable nature of when the stop sign was displayed (1–60 seconds after the first 3 minutes of driving) only the first 3 minutes (180 seconds) of driving were used for analysis of the effects of the images on speed of travel. This is in contrast to the 150 seconds of data used in the analysis of the psychophysiological measures mentioned in section 2.2.3 above

The dependent measures analysed in this experiment were actual speed driven, stopping reaction time, the subjective ratings of speed, effort and risk, performance on the memory task, and the psychophysiological measures of heart rate, heart rate variability, skin conductance and respiration. In addition qualitative data on differences between the roads and on whether the participants noted anything unusual were also examined.

Using PASW SPSS 18.0.3 for Windows, individual full factorial repeated measures analyses were carried out to examine the effect of the independent variable of the target image type within the subjects (2 levels, emotionally negative or neutral target image). In addition the between subjects factors of condition order (2 levels, neutral image presented first or negative image presented first) and gender were also examined. Possible interactions effects were examined and post-hoc tests with a Bonferroni correction were used where appropriate. The above analyses were run for both the total dataset, and separately for the males and females.

## Results

### 3.1 Subjective ratings of speed, effort and feeling of risk

In general the participants gave higher ratings for effort than for feeling of risk, with average scores between 53.77 and 59.12, corresponding approximately with a level of ‘Rather much effort” on the RSME. Whereas feeling of risk scores averaged between 30.50 and 34.93, placing them somewhere between ‘A little risk’ and ‘Some risk’ on the modified version of the scale. As shown on table 1 there was no significant main effect of the target image type (negative or neutral) on subjective ratings of speed, typical travel speed, effort, or feeling of risk.

**Table 1:** Subjective ratings of speed, effort, and feeling of risk by target image type, gender, and condition order for the combined dataset (N = 85).

	Negative then Neutral order				Neutral then Negative order			
	Negative		Neutral		Neutral		Negative	
	Mean	SD	Mean	SD	Mean	SD	Mean	SD
Subjective Speed (km/h)	60.41	20.53	69.88	20.66	60.69	18.21	72.50	21.02
Typical Speed (km/h)	92.33	15.97	94.07	15.78	88.81	18.04	90.36	15.94
Effort	59.12	24.10	55.62	25.38	57.52	24.83	53.77	26.20
Feeling of risk	31.73	25.92	34.93	26.46	30.50	25.09	34.36	24.71

	Statistical tests																	
	Main effects									Interaction effects								
	Image			Gender			Order			Image * Order			Image * Gender			Image *Order*Gender		
	F	p	η^2^	F	p	η^2^	F	p	η^2^	F	p	η^2^	F	p	η^2^	F	p	η^2^
Subjective Speed (km/h)	1.94	0.17	0.02	1.45	0.23	0.02	0.18	0.68	0.00	42.87	<.001	0.35	1.3	0.26	0.02	2.36	0.13	0.03
Typical Speed (km/h)	0.17	0.68	0.00	3.89	0.052	0.05	1.06	0.31	0.01	3.05	0.09	0.04	0.7	0.41	0.01	0.02	0.88	0.00
Effort	0.39	0.54	0.01	0.38	0.54	0.01	0.50	0.48	0.01	8.54	<.01	0.10	2.26	0.14	0.03	1.4	0.24	0.02
Feeling of risk	1.43	0.24	0.02	2.05	0.16	0.03	0.04	0.84	0.00	1.40	0.24	0.02	4.15	<.05	0.05	4.16	<.05	0.05

There was however a significant interaction of target image type and condition order for the subjective ratings of travelling speed (F (1, 81) = 8.21, p<.001, η^2^ = .35) with the second trial generally resulting in higher average perceived speeds. In combination with this, trials where the negative target image condition was second resulted in larger increases in ratings of subjective travelling speed between the two drives. This indicates that the participants perceived a speed increase between the first and second drive, but perceived it as being a greater increase if they had been exposed to the negative image in the second, rather than first, trial.

A significant (F (1, 81) = 4.16, p<.05, η^2^ = .05) interaction of target image type, gender and order was also found for ratings of feeling of risk. This appears to have resulted from the males tending to give higher average ratings of feeling of risk for the negative target images than the females (40.72 or 41.70 on average for the males compared with 28.30 or 30.82 for the females). This also points to a different effect in terms of the order of image presentation, in that the males appear to decrease ratings of risk from the first to the second trial by 6.64 points if the negative target images were presented first, and increase ratings of risk by 3.86 if the negative target images were presented second. Conversely the females always increased their ratings of feeling of risk from the first to the second drive, and did so more if the neutral target images were presented second (an increase of 6.59 versus an increase of 3.86 if the negative target images were second).

There was also a significant (F(1,81) = 8.54, p<.01, η^2^ = .10) order and target image type interaction on ratings of effort, with ratings of effort generally decreasing in the second trial, but doing so more if the negative target images were second (a decrease of 3.75 compared with a decrease of 3.50 if the neutral target images were second). However if the male and female datasets are examined separately then this significant order and target image type interaction is only apparent in the male data (F (1, 24) = 6.19, p<.05, η^2^ = .21) and is different from the combined dataset. In that the decreases in effort ratings from the first to second drive are still apparent in the male data set but are greater when the neutral target images were second (a decrease of 9.76 compared with a decrease of 3.10 when the negative target images were second). No significant main (F (1, 57) = .64, p = .43, η^2^ = .01) effects of the images or interaction effects (F(1, 57) = 2.48, p = .12, η^2^ = .04) with order on ratings of effort were found for the female participants However, the females did tend to also decrease effort ratings from the first to the second trial, and did so somewhat less if the neutral images were second (a decrease of 1.35 compared with a decrease of 4.11 if the negative images were second), although again this effect was not significant.

When data from the males and females was examined separately then a main effect of target image type (F (1, 24) = 4.56, p<.05, η^2^ = .0.16) was found on ratings of typical speed for the males (N = 26), which is not in the combined dataset. In addition in the male dataset significant target image type and order interactions were found on typical speed (F (1, 24) = 7.45, p<.05, η^2^ = .24), subjective travelling speed (F (1,24) = 8.87, p<.01, η^2^ = .27), and effort (F (1,24) = 6.19, p<.05, η^2^ = .21). Finally in the male data there was no significant main effect (F (1, 24) = 2.81, p = .81, η^2^ = .11) nor any interaction effects with order (F (1,24) = .20, p = .66, η^2^ = .01) of target image type on feelings of risk. In case of the above effects it seems that the males tended to report higher subjective travelling speeds in the second trial, although more so when the negative target images were second. With an increase of 11.07 km/h on average compared with an increase of 3.64 km/h if the neutral target images were second. The impacts in terms of effort are discussed above, with lower effort being generally reported in the second trial, but more so if the neutral target image trial was second. The effects seen for typical speed seems to have arisen because the male participants who received the neutral and then negative target image trial order on average only increased their ratings of typical speed by 0.33 km/h between trials. Whereas those who experienced the neutral and then negative target image trial order increased their ratings by 2.73 km/h between the first and second trial. This result is unlikely to be meaningful.

In terms of the results for the females (N = 59), they were similar to those of the total sample, with significant target image and order interactions for subjective travelling speed (F (1, 57) = 54.37, p<.001, η^2^ = .49) and feeling of risk (F (1,57) = 9.98, p<.01, η^2^ = .15). Again, it seems that for the females their perceptions of the subjective travelling speed increased from the first to the second drive with larger increases when the negative target images were presented second (12.22 km/h versus 11.48 km/h when the negative images were first). As described above, the effect on feeling of risk for the females was an increase between the first and second drive, with a larger increase if the neutral target image was second (6.59 versus 3.86 if the neutral target image was first). As mentioned above no significant main or interaction effects of the target image type or order were found for ratings of effort in the female dataset. As with the combined dataset there were also no significant main (F (1, 57) = .11, p = 0.74, η^2^ = .74) or interaction (F (1, 57) = 2.16, p = .15, η^2^ = .04) effects of the target images on ratings of typical speed either.

### 3.2 Stopping reaction time

There was no significant (F(1,81) = 2.98, p = .09, η^2^ = .04) main effect of target image type on stopping reaction time in reaction to the stop sign, with an average reaction time of 2.04 (SD = 1.03) seconds for the negative target image trial and 1.87 (SD = .61) seconds for the neutral target image trial. There were also no significant interactions or main effect of order or gender (F (1,81) = .12 to 2.59, p>.11, η^2^ = .00 to .03).

### 3.3 Driving Speed

As shown in table 2 there was no main effect of target image type (F (1, 81) = 1.69, p = .20, η^2^ = .02) on average speed driven during the first 3 minutes of the experimental trials. There was however a significant main effect of gender (F (1, 81) = 8.21, p<.01, η^2^ = .09), with males tending to drive faster on average (114.61 km/h) than the females (96.47 km/h) across all trials.

**Table 2:** Average speed by target image type, gender and condition order for the combined dataset (N = 85).

	Negative then Neutral order				Neutral then Negative order			
	Negative		Neutral		Neutral		Negative	
	Mean	SD	Mean	SD	Mean	SD	Mean	SD
Speed (km/h)	98.65	31.16	105.72	29.68	99.10	24.34	102.53	27.39

	Statistical tests																	
	Main effects									Interaction effects								
	Image			Gender			Order			Image * Order			Image * Gender			Image *Order*Gender		
	F	p	η^2^	F	p	η^2^	F	p	η^2^	F	p	η^2^	F	p	η^2^	F	p	η^2^
Speed (km/h)	1.69	0.20	0.02	8.21	<0.01	0.09	1.31	0.25	0.02	20.25	<.001	0.20	0.65	0.42	0.01	0.48	0.49	0.01

There was also a significant interaction effect of target image type and condition order (F (1, 81) = 20.25, p<.001, η^2^ = .20). This means that speed during the second trial tended to be higher than during the first, but also that this effect did not appear to be as pronounced when the negative target images were presented to participants second. Specifically, when the negative target images were second there was an increase of only 3.43 km/h on average from the first to second trial compared with a 7.08 km/h increase in speed when the neutral target images were second.

**Figure 3:**
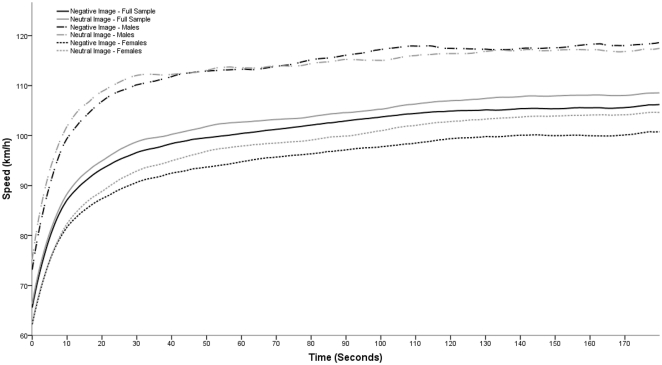
Average speed across the first 3 minutes for the negative and neutral target image trials. Lines are given for the whole sample (N = 85), as well as for the males (N = 26) and females (N = 59) separately.

As shown in figure 3 when the speed data for the males and females was examined separately there were quite different outcomes. In the case of the males (N = 26) it appears that there was no significant difference (F (1, 24) = .06, p = .81, η^2^ = .00) in the speed they drove when exposed to either the negative (113.29 km/h on average) or neutral target images (113.19 km/h on average). Neither was there a significant interaction of target image type and order (F (1, 24) = 3.62, p = .07, η^2^ = .13) for the males. Conversely if only the females (N = 59) were examined, then a significant main effect of target image type was found (F (1, 57) = 4.51, p<.05, η^2^ = .07) along with a significant interaction of target image type and order (F (1, 57) = 27.33, p<.001, η^2^ = .32). Therefore in the case of the females it seems that, on average, the trials with the negative images resulted in lower driving speed (94.96 km/h on average) than trials with the neutral images (97.72 km/h on average). Furthermore, as with the total sample, it appears that females tended to increase their speed from the first to second drive, but did so to a lesser extent if the negative target image trial was experienced second. In the females, this resulted in an increase of 7.91 km/h when the neutral target images were second, compared with an increase of 3.34 km/h when the negative images were second. As shown in figure 3 the speed difference between the negative and neutral target image conditions in the females began to become apparent after less than 10 seconds of driving, and is pretty much established by 20–30 seconds into the drive and then remains relatively constant. When looking at figure 3 this same initial speed pattern does seem to appear for the males, but quickly disappears, with some later average speeds for the negative target image trial exceeding those of the neutral target image trials. This is not statistically significant however.

### 3.4 Heart rate, heart rate variability, respiration and skin conductance response

All recorded psychophysiological measures were compared between the two target image trials, but also to the baseline measurement as a third variable. Furthermore, as with the above analyses, condition order and gender were included as between subject factors. It should be noted that, as explained in section 2.2.3, the analysis of the psychophysiological data was carried out on only 150 seconds of data and with a smaller sample size of 65 participants.

Significant (F (2, 60) = 19.16 to 50.02, p<.001, η^2^ = .39 to .63) main effects were found on average heart rate, as well as mid and high band heart rate variability. However post hoc tests with a Bonferroni correction revealed this was only due to significant differences between the baseline measurement and the measurements collected during the target image trials (p<.001). This means that no significant differences in these measurements between the target image trials was found (p = 1.00).

There was also a significant (F (2, 60) = 5.85, p<.01, η^2^ = .09) main effect of gender on mid band heart rate variability, with males having a higher variability than females on average. However, there were no interactions of the target image type with gender (F (2, 60) = .07, p = .92, η^2^ = .00) or condition order (F (2, 60) = .35, p = .71, η^2^ = .01) for mid band heart rate variability.

A significant (F (2, 60) = 5.12, p<.01, η^2^ = .15) main effect of target image type on skin conductance was found with the experimental trials seeming to produce a higher average skin conductance response than the baseline. However, subsequent post hoc tests with a Bonferroni correction only found a significant difference between the baseline measurement and the negative target image trial (p<.05) but no significant difference between the baseline measurements and the neutral target image trial (p = .07) nor between the target image trials themselves (p = 1.00). No significant effects for any of the variables or conditions were found for the respiration measurements.

There were also significant interaction effects between image type and condition order for average heart rate (F (2, 60) = 42.14, p<.001, η^2^ = .58) and high band heart rate variability (F (2, 60) = 5.02, p<.01, η^2^ = .14). Meaning that the average heart rate decreased and the average high band heart rate variability tended to increase in the second trial. As can be seen in table 3 the average decrease in heart rate was smaller between the first and second trials when the negative target image trial was second (a decrease of 6.39 beats per minute versus 6.82 when the neutral target image trial was second), and the average increase in high band heart rate variability was higher in these conditions (an increase of 0.34 versus 0.31 when the neutral target image trial was second).

**Table 3:** Average heart rate, heart rate variability, skin conductance response and respiration amplitude by target image type, gender and condition order for the combined dataset (N = 65).

	Negative then Neutral order						Neutral then Negative order					
	Baseline		Negative		Neutral		Baseline		Neutral		Negative	
	Mean	SD	Mean	SD	Mean	SD	Mean	SD	Mean	SD	Mean	SD
HR (bpm)	79.92	11.39	90.05	11.15	83.22	10.35	79.38	12.80	89.27	11.43	82.89	12.05
Mid HRV (mi^2^)	6.94	0.87	6.22	0.89	6.27	1.20	7.48	1.18	6.51	1.00	6.60	1.05
High HRV (mi^2^)	7.32	0.92	6.36	0.80	6.66	0.90	7.78	1.24	6.64	1.00	6.98	1.11
SCR (mho)	0.16	0.29	0.23	0.28	0.16	0.35	0.07	0.08	0.23	0.32	0.19	0.18
Respiration amplitude	1.04	0.70	0.72	0.52	0.75	0.62	1.89	4.81	1.03	1.39	0.87	1.45

	Statistical tests																	
	Main effects									Interaction effects								
	Image			Gender			Order			Image * Order			Image * Gender			Image *Order*Gender		
	F	p	η^2^	F	p	η^2^	F	p	η^2^	F	p	η^2^	F	p	η^2^	F	p	η^2^
HR (bpm)	44.19	<.001	0.60	0.06	0.82	0.00	0.06	0.81	0.00	42.14	<.001	0.58	0.05	0.95	0.00	0.16	0.86	0.01
Mid HRV (mi^2^)	19.16	<.001	0.39	5.85	<.01	0.09	3.64	0.06	0.06	0.35	0.71	0.01	0.07	0.92	0.00	0.39	0.70	0.01
High HRV (mi^2^)	50.02	<.001	0.63	1.13	0.29	0.02	1.63	0.21	0.03	5.02	<.01	0.14	0.26	0.78	0.01	0.03	0.97	0.00
SCR (mho)	5.12	<.01	0.15	0.57	0.45	0.01	0.01	0.92	0.00	2.43	0.10	0.08	0.70	0.50	0.02	1.64	0.20	0.05
Respiration amplitude	1.49	0.23	0.05	0.86	0.36	0.01	0.96	0.33	0.02	0.34	0.72	0.01	0.05	0.95	0.00	0.42	0.66	0.01

When the male (N = 22) and female (N = 43) participants were examined separately then similar results to those mentioned above were found, with two exceptions. The first being a significant (p<.05) difference for the males between their average baseline respiration measure and the average respiration measure during the negative target image trial. There was however no significant difference in respiration for the males between the baseline measures and the measures during the neutral target image trial (p = .20), nor any significant difference between the average respiration in the neutral and negative image trials (p = .69). The second difference is that for the females there was no main effect of condition (baseline, negative, or neutral target image) on skin conductance response at all (F (2, 40) = 2.47, p = .10, η^2^ = .11).

### 3.5 Memory task accuracy

While the memory task was simply an excuse to have images presented on the screen, it is worth noting that there was no significant effect of the type of target image (negative or neutral) on the number of pairs reported. Rather the participants performed this task well in both conditions with an average reported number of image pairs of 11.67 (SD = 3.15) during the negative target image trials and 11.49 (SD = 3.81) during the neutral target image trials. The actual number of image pairs was 12.06 (SD = 2.84) and 11.33 (SD = 2.79) for the negative and neutral target image trials respectively. This indicates that the ‘memory task’ was equally demanding during both conditions, and that the participants were paying attention to the images. If the male and female participants are examined separately then their performance on this task and the average number of images presented to them is similar to the combined dataset.

### 3.6 Reported differences between the roads

At the end of the experiment participants were asked ‘What was/were the difference(s), if any, that you noticed between the first and second roads you drove?’ This question, along with a question about the images, was primarily to check if the participants had seen any of the target images as the road did not differ between the two drives. However, only 43.53% (11 male, 26 female) of the participants correctly reported that there was no difference between the roads. A further 38.82% (9 male, 24 female) reported that something about the construction of the road had differed. Out of these people, 19 (7 male, 12 female) stated that either the road in the negative image trial was more curvy (12; 4 male, 8 female) or that the road in the neutral image trial was less curvy (6; 3 male, 3 female) (or in the case of one female that the roadside trees were further away during the neutral trial). Conversely, 14 (2 males, 12 females) participants said that either the road in the neutral image trial was more curvy (9; 1 male, 9 females) or had more hills (1 female), or that the road in the negative image trial was less curvy (4; 1 male, 3 females). In nearly all of the cases (18; 5 male, 13 female), if a road was labelled as more curvy it was the road that was driven second. Therefore it is likely that this impression of more or sharper curves was created by the fact that participants tended to drive faster during the second drive. In answer to this question, a further 9 (3 male, 6 female) participants commented that they had detected more image pairs during the ‘memory task’, or that the images were placed in a different position on the second drive (1 female). Of the remaining 4 participants, 2 did not answer this question, and the other 2 just mentioned being more used to the road on the second drive.

## Discussion

According to many models of driver behaviour [1]–[10] our feelings and emotions play important functional roles in guiding driver behaviour. What is more, it is claimed that this can occur even when emotions aren't necessarily felt or explicitly considered as part of the decision making process. The present study set out to investigate the impact of these implicit or unconscious emotions on driving behaviour. As such, masked images were used in an attempt to provoke an emotional response in drivers in a simulator under the guise of performing a memory task.

The primary behavioural variable examined was driving speed, and if the total dataset of males and females is examined then there was no significant main effect of the target image type on driven speed. However, there was a significant interaction effect of type of target image, negative or neutral, and the order in which the images were presented to the participants. This showed that there was a general tendency for participants to drive faster on the second trial, most likely due to familiarity and learning effects. However this general effect also interacted with the type of target image being used, with the increase in speed being smaller if the second trial contained the negative target images (an increase of 3.43 km/h on average) than if the neutral target images were presented second (an increase of 7.08 km/h). This suggests that the negative target image could have had a suppressing effect on speed in terms of reducing the normal increase associated with familiarity or learning effects.

Gender also played a role in this effect, and when the male and female datasets were examined independently then was no effect, interaction or otherwise, of the target images on the males speeds. However for the females there was the above interaction effect mentioned for the combined dataset, and also a further significant main effect of image type on driving speed. This resulted in significantly lower average speed overall for the female drivers during the negative target image trials (94.96 km/h) than during the neutral target image trials (97.72 km/h). This gender difference is apparent in the statistics and also easy to see from the graph of speed over time shown in figure 3 and suggests that most, if not all, of the effects seen in terms of actual driven speed in the larger combined dataset are due to the reactions of female participants.

The difference between the male and female participants could be explained in several ways. Firstly it is possible that the negative target images were more emotionally impactful for the female participants than the males. Certainly there is a difference in both valence and arousal ratings by gender for many images in IAPS [49], and specifically if the negative target images used in this experiment are examined, then the female IAPS ratings are lower in valence (1.52 on average) and higher in arousal (6.98 on average) than the average provided by the male IAPS ratings (2.16 valance, 6.15 arousal on average). An increased reactivity to negative emotional images in females is also supported by studies reporting greater neurological [53], [54] and autonomic reactions [55], [56] to explicitly presented negatively emotional images in females. This is especially so if the negative images contained humans [57] as many of the images used in the current experiment did. It is entirely possible therefore that the increased emotional reaction to consciously or explicitly processed stimuli in females may also hold for stimuli that have been implicitly or preconsciously processed. If this is so further studies should use different image sets depending on the gender of the participant.

Another possible explanation for the gender difference is that given the effect sizes observed that there may not been enough males to detect any consistent effect of the images on behaviour. This study did set out to recruit a large number of both male and female participants to take part; however we did not succeed in the case of the male subjects.

Interestingly, at least in the case of the females, the effect of the negative target image on driving speed seems to be mainly on the initial setting of the speed, which is then generally maintained throughout the drive (see figure 3). This pattern of a slower average driving speed for the negative image occurring near the start of the drive does also seem to appear briefly for the males, but then disappears quickly as the drive continues. The fact that the effect on driving speed occurs so quickly could be taken as support for the assertions of various models [1]–[10], [16], [21], [58] that driver behaviour is influenced by a tendency to return to or maintain some kind of homeostatic body balance or preferred safety margin. Unfortunately it cannot throw light onto the differences between these models, in terms of suggesting whether this body balance is itself constantly monitored and a set level targeted [1]–[3], [5]–[8], [16] or if it is arrived at through an aversion to signals that arise because of a unbalance in this body state [4], [10], [21], [22], [59].

Moving away from speed to the recorded psychophysiology there is a significant target image type and order interaction effect in both heart rate and high band heart rate variability that is potentially interesting. This interaction effect is found in the combined dataset, in the males and, most importantly in light of the above speed effects, in the female dataset. The interaction indicates decreases in heart rate, and increases in high band heart rate variability between the first and second drive. This is consistent with a familiarity or relaxation effect with the second drive becoming less stressful or effortful for the participants. Interestingly however, the decrease in heart rate and increase in high band variability was lessoned if the negative target images were second. This, when combined with the above speed data where the increase in speed was also less when the negative images were second, is suggestive of a physiological effect of the negative target images. Specifically it appears that the usual familiarity effect in terms of increasing speed, but also in terms of decreased physiological load for the second trial was lessoned if the negative images were presented second. That this effect is seen in the cardiovascular measures but not in skin conductance, which does not seem to meaningfully differ between the trials, or in the case of the female participants even between the baseline and the driving task, is interesting. While both cardiovascular and skin conductance measures are sensitive to changes in emotional arousal, heart rate and high band heart rate variability are generally considered to be more reflective of changes in mental workload or effort [52], [60]. This may imply that the negative target images used are actually impacting on the mental workload or effort required by the task rather than creating feelings or emotions of risk, and it is the physiological reaction to this increase in effort that leads to the reduction in driving speed. If so this would be more in line with predictions made by models such as the multiple comfort zone model [4], [10] or task difficulty homeostasis [1], [2] which claim that emotions and feelings associated with the difficulty or effort required in the driving task are more common guides of driver behaviour than emotions related to risk.

Care should be taken with this interpretation however. Another way to interpret the lack of an impact on skin conductance response would be to say that because the participants (at least the females) drove, on average, slower when influenced by the negative image they were bringing their body state back into its normal range. This process of returning to a normal, comfortable, or set body state may occur quite quickly, during the first few seconds of driving, and therefore may not show up in the averaged psychophysiological data presented here. Especially since the first 30 seconds of psychophysiological data had to be excluded from analysis. This data was excluded because there was an immediate large impact of beginning to drive and starting to perform the memory task on the cardiovascular and skin conductance measures. The above mentioned physiological consistency or homeostasis is what would be predicted by models of driver behaviour such as RAT [3] or RMM [5]–[7] which more closely embrace the work of Taylor [16]. Therefore as already mentioned it is difficult to use this data to distinguish between the competing models in traffic psychology.

A related explanation for the lack of a meaningful response to the target images in skin conductance could be due to ceiling effects related to performing the driving task. Previous studies which have shown an effect of masked images on psychophysiology have tended to involve simple experimental tasks carried out in a standard lab environment [28]–[30]. This relatively simple task environment means that any physiological impact will be easier to detect. Furthermore, the physiological impacts of the masked images reported in these previous studies are often relatively small. On the other hand, simply driving has a large impact on psychophysiological measures, even with the simple road design used in this study [61]. This driving task related impact may therefore have masked detection any physiological effect of the emotional images. This however does make it even more significant that the order effect on heart rate and high band heart rate variability discussed above was found.

It was also thought that the negative image may have primed the participants to be aware of any possible threats and made them ready for action, resulting in a faster reaction time in response to an image of a stop sign being presented on the screen. However, no such effect was found in the combined, male, or female datasets. This could again be explained through the above mentioned return to a normal body state at the start of the drive. If participants had indeed quickly eliminated or balanced out the effect of the negative image on their body state by lowering their speed, then the negative image would possibly have no further impact, and could not help in raising their alertness to the onset of the stop sign. Another explanation could be that the stop sign in itself, while a road safety related stimulus, is not threatening enough when it occurs in an open road situation with no other traffic. Therefore perhaps future experiments investigating the influence of masked negative images on threat detection could use a more relevant stimulus such as a car unexpectedly pulling out from a parking spot.

There was also a significant interaction effect of target image and order on participant's perceived speed in the combined, male, and female datasets. This reflects that actual travel speeds increased between the two trials. However, in this case the perceived increase in speed was larger if the negative images were presented during the second trial (a perceived average increase of 11.8 km/h compared to 9.48 km/h if the neutral target image was second). At least for the female participants this is the reverse of the effect on actual driven speed, with the speed increase in this case being smaller when the negative target images were presented second. This could be interpreted as exposure to the negative target images creating an impression of faster driving speeds and therefore influencing the speed actually driven. However, it is clear from the data that this difference in speed perception, while present in the female participants, is mostly contributed to by the male participants who did not seem to be significantly affected by the target images in terms of their actual driving speed.

The results for the subjective ratings of feeling of risk and effort are somewhat difficult to interpret for the combined dataset. In the case of ratings of risk there is not only a significant interaction of target image type and order, but also a significant interaction with gender. This means that in the case of the male participants they decreased their ratings of feeling of risk from the first to second trial if the negative target images were presented first (a decrease of 6.64 on average), but increased their ratings if the negative target images were second (an increase of 3.86 on average). However, if the male dataset is analysed separately there are no significant target image or order effects or interaction on ratings of feeling of risk, which makes it difficult to draw any conclusions from this finding. Conversely if only the female participants are examined there is a significant target image type and order effect with a tendency to increase ratings of risk between the first and second drives, with larger increases occurring if the neutral target images were second (an average increase of 6.59 compared with an increase of 3.86 if the negative target images were second). This again is difficult to explain, but perhaps could be related to the participants, correct, perception that speeds were increasing from the first trial to the second. It is therefore possible that participants rationalised that since they were driving faster, then the risk must also be greater. Although why this effect occurs significantly more in the presence of the neutral image for the females is not clear. However, it does at least in the case of the female participants suggest that the emotionally negative images were not having any significant increasing effect on the female participant's feelings of risk.

The situation in the case of ratings of effort differs from that for ratings of feeling of risk. In in the combined dataset there is a significant order and target image type interaction, with ratings of effort generally decreasing between the first and second trials, and this effect seems to be larger if the negative target images were second (a decrease of 3.75 compared with a decrease of 3.50 if the neutral target images were second). However, if the male and female datasets are examined separately it appears the majority of this effect comes from the male participants, who in contrast to the combined dataset and the female participants, decrease their ratings of effort more if the neutral target images were second. In the case of the female participants their data appears similar to that for the combined dataset, although there were no significant main effects or interactions.

The above findings for ratings of effort are particularly interesting in light of the previously discussed impacts of the target images on perceived speed, actual speed driven and on the psychophysiological measures of heart rate and high heart rate variability. In this case the target image type and order interaction effect observed in the other variables is reversed in the combined dataset for ratings of effort, or in the case of the females is non-significant. This, when combined with no consistent effect on ratings of subjective feelings of risk do seem to suggest that the participants subjective feelings were not altered by the images, despite producing the observed effects on speed, perceived speed and psychophysiology.

Finally, when asked about the differences between the roads they had driven only 43.53% of the participants correctly identified that the roads had not differed. If the remaining participants are examined then 38.82% reported something had changed about how the road was constructed. Typically participants mentioned that the second road they drove had more curves. The rest of the participants either commented on the memory task, suggesting that they had seen more pairs on one of the trials, or did not answer this question. That some participants did report a difference in the road is likely due to the demand characteristics of being in an experiment and being asked if there were differences. Having been asked, they perhaps felt pressured to say that there were. It is interesting however that this resulted in the second drive being generally attributed the characteristic of being more curvy, and this is likely to do with the fact that the second road tended to be driven at a higher speed. A similar finding was made by Lewis-Evans and Charlton [62] where drivers ascribed risky characteristics such as heavier traffic, missing road marking and more curves to a simulated road that had been narrowed by 2 meters, but was otherwise identical to other roads they had driven.

Apart from the points already made above there are several other potential issues that can be raised with this study. One obvious issue is the question of whether the participants really were unaware of the images. As mentioned in the introduction, this is a controversial issue [25]–[27], and therefore care has been taken to ensure that at the very least our participants could not explicitly report having seen the images. As such during the setup of the experiment the timing of the image presentation in the simulator was confirmed over three separate four minute periods using a high speed camera. Also a manipulation check question was included asking participants if they had noticed anything unusual about the images and they were also debriefed after the experiment and asked if they had seen any of the images. Based on this manipulation check, two females and six males who explicitly reported seeing the images were removed from the study, and data from an additional seven males and 12 females were removed on the grounds that they mentioned that maybe they saw something in between the masking images. These last 19 participants generally made statements along the lines of ‘perhaps there was an image between the ones I saw, but I could not tell you what’ or ‘I think I saw flashes of colour’. The removal of these participants means that the remaining data is only from participants that explicitly stated that they did not see anything unusual with the images, and did not at any time during the experiment, including during the debrief, mention explicit awareness of the target images nor any suspicion that a masking procedure was occurring. As such we are relatively confident that the participants were not explicitly aware of the target images. Future studies may however want to consider using a forced recognition task for each subject as part of the experiment in order to confirm that participants were completely unable to consciously perceive the target images.

A greater proportion of males (15.38%) explicitly saw the target images compared to the female participants (2.78%). Although a similar proportion of males (17.95%) and females (16.67%) were in the group that was removed for suspecting that masking was occurring. This gender difference could be related to the generally better visuospatial abilities typically attributed to males [63], [64]. Also, anecdotally, those most males who explicitly saw the images also reported during the debriefing period that they considered themselves to be gamers. Since video games are also suggested to improve visual attention [65]–[68], particularly for fast moving or rapid stimuli, this may also be a contributing factor in noticing masked images.

Another issue is with the order effect that was encountered. The general tendency for participants to increase their speed from the first to second trial is not uncommon in simulator research [69], but could still be of concern. On one hand this may have been because the practice time given to the participants was not sufficient for them to become comfortable with the memory task and simulator. However, as reported in section 2.3, participants were asked if they felt comfortable with the memory task and the simulator after the practice drive and offered the option of repeating the task if they felt it was necessary. None took this opportunity. Nevertheless, this could be addressed in future studies through the provision of a longer practice period. It is also worth noting that the order effects found where always in interaction with the target image variable, and that there were no main effects of the trial order alone on any of the variables.

In addition to a longer practice period, longer experimental driving trials could also be investigated. These could perhaps include changes from emotionally neutral to emotionally negative image targets occurring on several occasions during the drive itself. This would also allow for the psychophysiological data to be collected for longer periods, and reduce the problem of having to remove the initial increase in these variables caused by simply starting to drive. All this said, at least in the case of the female participants, the effect of the negative target images on speed behaviour appears to occur within the first 20 seconds or so after the images have been presented and then maintained overtime. Therefore the time periods used in this experiment do appear sufficient to catch at least this critical moment in terms of the impact of the target images on driving speed.

Another potential issue is the high level of variability in the speed data. This was likely caused by the fact that participants were denied the use of a speedometer and told to drive at whatever speed they were most comfortable at, resulting in large between subjects differences in speed. However, due to the nature of the within-subjects repeated measures analyses used in this study the large variability between the subjects should not have had a negative impact on the results.

The fact that the participants were denied the use of the speedometer could also challenge the validity of this experiment. The speedometer was removed in an attempt to force participants to rely on their own perception of speed, and therefore hopefully encourage them to use the automatic or implicit control processes that are most likely to be affected by the masked target images. Furthermore it allowed for the question about participants perceived speed of travel to be asked. Still, the lack of a speedometer is not a common occurrence in every day driving. However, evidence from studies of drivers gaze patterns show that they typically devote very little time, as low as 0.6% of total gaze time during a drive, glancing at their speedometers [70]. This indicates that most of the time drivers on the road are relying on their own perception of speed, not information from the speedometer, much like they had to in this experiment.

The type of target images used could also be questioned, especially in terms of the negative target images. In the case of this study the images were selected as being some of the lowest valence and highest arousal negative images in IAPS, along with four motor vehicle accident related images. This means that the majority of the negative target images were of mutilated and deceased humans removed from the typical driving context. These IAPS images were used as they were of a known quality, taken from an internationally recognised sample, and had been previously used in masking studies [30], [44]. As such this experiment should be taken as a proof of concept. Future studies could perhaps use more driving relevant stimuli, such as risky traffic situations or images related to the presence of police enforcement.

The validity of the “memory task” that the participants were performing in terms of normal driving could also be challenged. It is indeed a somewhat unusual task to be paying attention to an additional visual element and trying to detect repeated patterns in it while driving. However driving is a visual task, and often does require that visual attention be split, and that changes or the lack of change in the visual environment be detected and remembered. For example it may be important to recognise landmarks and know if you have seen them before when trying to navigate from A to B. Also given that the same task was required of all participants, and seems to have been consistently well performed, it is unlikely that it impacted on the results of the experiment in any meaningful way.

Ultimately it does appear that the masked negative target images did have an impact on the behaviour of the participants in this experiment, at least in the case of the females. This seems to primarily have occurred via suppression or a lessening of the normal familiarity effect between the first and second experimental drives when the negative target image was shown in the second drive. The data from the psychophysiological measures in terms of heart rate and high band heart rate variability support this interpretation and when coupled with the lack of any meaningful effect on subjective ratings of effort or risk suggests that the impact of the negative target images was not explicit. Interestingly there does seem to be one clear subjective effect, with the participants perceiving the second drive as faster in general, but more so when the negative target image was second. This therefore suggests an impact of the negative target image on speed judgement, although care should be taken with this interpretation as the majority of this effect seems to be amongst the male, rather than the female, participants.

The explanations given above for the results of this study could be labelled as speculative. However, this study does represent the first attempt to use masked images as part of a driving task, which in itself is noteworthy. We are unaware of any other studies examining the effect of masked images on a behaviour as complex as driving, which just by itself can have a large impact on psychophysiology [40]. Therefore the very fact that any effect in behaviour was found despite the usually somewhat small changes in physiology in reaction to masked images reported by other studies [15], [16], [17] is significant. That the masked images can produce an effect on such an important variable for road safety as speed suggests that the influence of implicit or unconscious emotions on driver behaviour should be further studied. With this in mind, the results of this study are an interesting starting point, and can hopefully be used to help to guide future research.
